# Synergistic effect of essential oils and chlorhexidine against planktonic and biofilm-forming cells of *Malassezia pachydermatis*

**DOI:** 10.1016/j.vas.2024.100397

**Published:** 2024-09-10

**Authors:** Peter Váczi, Eva Čonková, Zuzana Malinovská

**Affiliations:** Department of Pharmacology and Toxicology, University of Veterinary Medicine and Pharmacy in Košice, Komenského, 73, 041 81, Košice, Slovakia

**Keywords:** *Malassezia pachydermatis*, Dogs, Essential oils, Chlorhexidine, Synergy

## Abstract

*Malassezia* (*M.*) *pachydermatis*, is often associated with secondary infection of the skin and external auditory canal in dogs and cats. The treatment of *Malassezia* infections is based on the local application of antifungals often combined with antiseptics. Due to increased resistance of yeast to commonly used antimycotics, especially in biofilm-forming cells, the use of natural substances, e.g. plant essential oils, appears as a new promised option. In this study, the efficacy of selected plant essential oils (EO) – oregano, rosemary, bergamot, clove, cinnamon, and thyme – in combination with chlorhexidine on both planktonic and biofilm-forming cells of *M. pachydermatis*, was investigated. The checkerboard test was used to determine the effect of chlorhexidine combined with individual EOs. According to the FICI (fractional inhibitory concentration index) in planktonic cells, most combinations showed additive effect, except for thyme and rosemary EO, where a synergistic effect was found (33.3 % and 16.7 % respectively). In the biofilm-forming cells, a synergistic effect was noted in chlorhexidine combined with bergamot EO, recorded in 6 isolates (33.3 %), and with thyme and oregano EO, detected in 3 isolates (16.7 %). A significant decrease (*p* ˂ 0.05) was found in FIC (fractional inhibitory concentration) compared to MIC (minimum inhibitory concentration), for both planktonic and biofilm-forming cells. Based on the obtained results, we can conclude that the combination of chlorhexidine with EOs achieved better efficiency than when using each agent alone and made it possible to reduce the concentration of both, and a sufficient antifungal and antibiofilm effect was achieved in *M. pachydermatis* strains.

## 1 Introduction

The lipophilic yeasts of the genus *Malassezia*, represent a part of the natural animal and human microbiota. They are also recognised as opportunistic microorganisms that can cause wide range of skin infections (Angiolella et al., 2023).

*Malassezia* (*M.*) *pachydermatis* is a significant zoophilic yeast, often isolated in dogs and cats, and is mainly associated with otitis externa and seborrheic dermatitis (Guillot & Bond 2020). Invasive infections caused by *M. pachydermatis* and lipid-dependent species can also occur in neonates receiving intravenous lipid supplementation and in immunocompromised patients who receive parenteral catheter nutrition (Velegraki et al., 2015).

*M. pachydermatis* possesses the ability to form biofilm *in vitro* and *in vivo*, thereby reducing its susceptibility to antimicrobial agents (Jerzsele et al., 2014). Biofilm formation, considered as one of the imporatnt virulence factors, enhances the pathogenicity of yeasts colonizing the host (Canizzo et al., 2007). A biofilm represent an immobile microbial community of cells strongly adherent to each other and to the biotic or abiotic surfaces. The cells of biofilm are protected by an extracellular matrix composed of polysaccharides and differ in phenotype from planktonic cells (Figueredo et al., 2013, Angiolella et al., 2023).

Antifungal treatment is generally successful in controlling yeast overgrowth, but occasionally it fails or relapses rapidly. The primary reason for this is considered insufficient identification and resolution of primary causes as well as predisposing factors. While the role of the antifungal resistance phenomenon is still not well defined, the scientific community has not yet adopted a common strategy for dealing with *M. pachydermatis* antifungal resistance especially a standard procedure for *in vitro* susceptibility testing (Peano et al., 2020).

Topic treatment by using the antiseptic substance is preferred when *M. pachydermatis* is located on the *stratum corneum*. There are several commercially available preparations containing 2 % to 4 % chlorhexidine which may be combined with azole antifungals. Topical treatment can reduce the cost and adverse effects associated with systemic antifungal treatment (Hoes et al., 2022). Chlorhexidine is a bis-biguanide compound that binds to the bacterial cell wall destroying cell membranes, and it is effective against yeast and fungi as a broad-spectrum antimicrobial agent (Coskun & Viskjer 2022).

In connection with raising resistance of yeasts to azoles, the interest in alternative topical antifungal drugs, such as plant essential oils, has increased. Essential oils (EOs) are concentrated, hydrophobic substances containing volatile aromatic compounds from different parts of plants (Pistelli et al., 2012). *Asteraceae, Lamiaceae, Myrtaceae, Rutaceae* and *Zingiberaceae* are the families mostly exploited to extract essential oils. These substances are secondary metabolites, characterized by the presence of mono- and sesquiterpenes including carbohydrates, alcohols, ethers, aldehydes and ketones, responsible of the distinctive smell and taste (Raut & Karuppayil, 2014). Most of the active compounds are terpenes, but also non-terpenoid compounds such as eugenol, cinnamaldehyde, and safrole possess biological activities (Donato et al., 2020). Numerous EOs produce pharmacological effects, exhibiting anti-inflammatory, antioxidant, and anticancerogenic properties (Kalemba & Kunicka, 2003).

In the field of veterinary medicine, EOs can be used as potential repellents against ectoparasites, as supplements with a positive effect on atopic dermatitis, chronic dermatitis, pyoderma, nasal hyperkeratosis, malodour, as ingredients in mouth rinses and to treat abscesses in dogs (Bismarck et al., 2019). Only a few *in vitro* studies report antifungal or antibiofilm activity of EOs against *M. pachydermatis* isolates (Khosravi et al., 2016, Váczi et al., 2018, Bismark et al., 2019) and the possible combination of EOs with antifungals (Bohmova et al., 2019, Schlemmer et al., 2019).

The main goal of this study was to found out the antifungal and antibiofilm efficiency of selected plant essential oils in combination with chlorhexidine against clinical strains *of M. pachydermatis* and to compare their activity with the effect of tested agents used alone.

## 2 Material and methods

### 2.1 Samples of *Malassezia pachydermatis*

The experiments were realised on 18 clinical isolates of *M. pachydermatis*, obtained in cooperation with the Small animal clinic at the University of Veterinary Medicine and Pharmacy in Košice, Slovakia. The samples were acquired from 18 dogs of different ages (3 months – 11 years), genders, and breeds (2 Cocker Spaniels, 1 Dachshund, 2 Irish Setters, 3 Maltese dogs, 3 Yorkshire Terriers, 1 Medium Poodle, 2 Golden Retrievers, 1 Labrador Retriever, 3 Crossbreeds). Dogs were diagnosed with *Malassezia* otitis or dermatitis. For the microbiological examination, samples were collected from the ears by swabbing the external ear canal and from the affected areas of skin by rubbing the skin with a cotton swab. The species identification was performed and confirmed based on phenotypic (macroscopic and microscopic) and genotypic characteristics (PCR-RFLP) described by Kaneko et al. (2007) and Gaitanis et al. (2002). *M. pachydermatis* CBS 1879 (CBS Utrecht, Holland) was used as a reference strain.

All the *M. pachydermatis* strains tested were biofilm producers, which was confirmed by methods described by Bumroongthai et al. (2016).

### 2.2 Methods used

The checkerboard assay according to Nikolić et al. (2017) was used to determine the susceptibility of both planktonic and biofilm-forming yeast cells to the tested agents.

### 2.3 Essential oils used

Six essential oils of clove (*Syzygium aromaticum*), cinnamon (*Cinnamomum zeylanicum*), thyme (*Thymus vulgaris*), oregano (*Origanum vulgare*), bergamot (*Citrus × bergamia*), and rosemary (*Rosmarinus officinalis*) (Calendula company, Nová Ľubovňa, Slovakia) were purchased for purposes of the present study. The certificate with the listed components detected by gas chromatography was included in each EO. Table 1 resumes the most important components of EOs used.

**Table 1:** Content substances of essential oils used.

Essential oil – plant species	Main content substances
Clove (*Syzygium aromaticum*)	eugenol (85.0 ± 3 %)
Cinnamon (*Cinnamomum zeylanicum*)	eugenol (77.0 ± 3 %)
Thyme (*Thymus vulgaris*)	*ρ*-cymene (40.0 ± 3 %), thymol (32.0 ± 2 %)
Oregano (*Origanum vulgare*)	carvacrol (85.0 ± 3 %)
Bergamot (*Citrus × bergamia*)	limonene (36 %), linalool (15 %), linalylacetate (23 %)
Rosemary (*Rosmarinus officinalis*)	1,8-cineole (25 %); α-pinene (19 %)

### 2.4 Testing the planktonic cells

#### 2.4.1 Preparation of planktonic cells

The 72-hour-old yeast isolates and reference strain were used to prepare a yeast suspension by mixing several colonies with sterile saline (0.9 % NaCl) supplemented with 0.1 % Tween 80. Using a densitometer, the suspension was adjusted to the density of McFarland 1, which corresponds to 10^6^ colony-forming units (CFU) in 1 mL. Subsequently, the suspension was diluted with Sabouraud's broth (HiMedia, Laboratories Pvt., Mumbai, India) containing 0.1 % Tween 80 (SBT) in a ratio of 1:100, which corresponds to a final concentration of 10^4^ CFU/mL.

#### 2.4.2 Dilution of essential oils and chlorhexidine

A 20 % solution (200 mg/mL) of chlorhexidine gluconate (Sigma-Aldrich, Merck Life Science, Germany) was used to prepare concentrations ranging from 5 mg/mL to 0.02 mg/mL, by dilution with SBT.

Similarly, 20 % solution of the tested essential oils was prepared in the form of an emulsion by adding gum arabic in the amount of 30 % of the essential oil volume. SBT was also used as a solvent in this case. Emulsion of each essential oil was prepared in the range from 50 mg/mL to 0.8 mg/mL.

#### 2.4.3 Checkerboard test – planktonic cells

The assay was performed in sterile 96-well microtitre plates with a U-shaped bottom. Columns 1 – 9 (within rows A – H) contained the decreasing concentration of chlorhexidine in a volume of 50 µl. Subsequently, the essential oil emulsion was added to the microtitre plate in descending concentrations, in rows A – G within columns 1 – 10, in the amount of 50 μL. Row H contained only the concentration gradient of chlorhexidine and column 10 only the concentration gradient of essential oil. By this step, both test substances were diluted by half. Afterwards, 100 μL of inoculum was pipetted into prepared microplates in columns 1 – 10 and in column 12 (positive control; containing 100 µl of inoculum and 100 µl of SBT). Column 11 served as negative control containing only 200 µl of SBT. By adding the inoculum, the following concentration gradients were obtained: for chlorhexidine in the range from 1.25 mg/mL – 0.005 mg/mL and for the essential oil from 12.5 mg/mL – 0.2 mg/mL.

One microtitre plate was used to test one strain of *M. pachydermatis* and the combination of chlorhexidine with one EO. To keep the same volume in all wells of the microtitre plate, 50 μL of SBT was added to row H and column 10.

The microtitre plates were incubated for 72 h at 35 °C and then the minimum inhibitory concentrations (MIC) of tested agents alone and in combination (FIC – fractional inhibitory concentrations) were read. For a better reading of the MIC/FIC results, 5 μL of 0.15 % resazurin dye (Sigma-Aldrich, Merck KGaA, Darmstadt, Germany) was applied to all wells of the microtitre plates, 12 h before reading the MICs.

To express the antifungal effect of the combination of two substances, the partial inhibitory concentration index (FICI) was calculated according to the equation:

$$FICI=\frac{FIC1}{MIC1}+\frac{FIC2}{MIC2}$$

where MIC1 is minimum inhibitory concentration of chlorhexidine alone, MIC2 (minimum inhibitory concentration of essential oil alone), FIC1 (minimum inhibitory concentration of chlorhexidine in combination with essential oil), and FIC2 (minimum inhibitory concentration of essential oil in combination with chlorhexidine).

Based on the FICI values, the effect of the tested combination was interpreted as follows: FICI ≤0.5 – synergistic, FICI > 0.5 <2 – additive, FICI ≥2 <4 – indifferent and FICI >4 – antagonistic.

### 2.5 Testing the biofilm

#### 2.5.1 Preparation of the biofilm

A full sterile loop (1 µL) of 72-hour-old yeast grown on SAT (Sabouraud's dextrose agar supplemented with 0.1 % Tween 80) was transferred into sterile Erlenmeyer flasks with 20 mL of SBT to allow multiplication. The flasks were incubated at 35 °C on an orbital shaker at 80 rpm for another 72 h. After that time, yeasts grown in SBT were centrifuged (at 90 × g for 10 min) and washed twice with 5 mL of phosphate buffer solution containing 0.1 % Tween 80 (PBS+T). In this way, the cells were cleaned from the nutrient medium, which could cause a false reaction.

The multiplied cells were used to prepare an inoculum suspension of 10^6^ CFU/mL by adding PBS+T and adjusting to the density of 1 McFarland, using densitometer (Biosan, Latvia). Preparation of biofilm was followed the procedure of Bumroonghthai et al. (2016), partially modified. In this case, sterile 96-well flat-bottom microtitre plates (Brand GMBH + CO KG, Germany) were used. A total 150 μL of inoculum was added into columns 1 – 10 and column 12 of the microtiter plate. Column 11 was considered the negative control (without substances tested and inoculum) and column 12 (the positive control) contained only inoculum. The plates were incubated at 80 rpm and 35 °C for 24 h on an orbital shaker, to allow yeast to adhere to the microtitre plate surface (adherence phase). After this phase, the inoculum suspension was aspirated and the wells were washed twice with 300 μL of PBS+T. Subsequently, 300 μL of SBT nutrient medium was applied to all wells and the microtiter plates were incubated on an orbital shaker (80 rpm) at 35 °C for 72 h, to allow the adhered cells to form a biofilm (biofilm-formation phase).

#### 2.5.2 Dilution of essential oils and chlorhexidine

To test the effectiveness of the combination of chlorhexidine and the tested EOs on biofilm-forming cells, the same concentrations as for planktonic cells of *M. pachydermatis* were used (for chlorhexidine in the range from 1.25 mg/mL – 0.005 mg/mL and for the essential oil from 12.5 mg/mL – 0.2 mg/mL, both dissolved in SBT).

#### 2.5.3 Checkerboard test – biofilm-forming cells

A total of 100 μL of decreasing concentration of chlorhexidine was applied to the biofilm-coated wells in columns 1 to 9 (within rows A – H). The concentration gradient of essential oils (100 μL) was added to rows A–G (within columns 1–10). The microtitre plates were incubated at 35 °C for 72 h on an orbital shaker at 80 rpm. Also in this case, 5 μL of 0.15 % resazurin dye solution (Sigma-Aldrich, Merck KGaA, Darmstadt, Germany) was added into the wells 12 h before reading the results. From the obtained MICs and FICs values, the FICI index was calculated in the same manner as for planktonic cells.

#### 2.5.4 Statistical analysis

The data are presented as average means (x̅), standard deviations (SD), mode (Mo) and median (Me). One-way ANOVA followed by Tukey's multiple comparisons test was used to analyse the mean of MICs (tested agents alone) and FICs (tested agents in combination) of the chlorhexidine and essential oils with each other and to compare the mean FICI in planktonic and biofilm forming cells of *M. pachydermatis* (GraphPad Prism 8.0.1, San Diego, CA, USA). The level of statistical significance was set up at *p* ˂ 0.05.

## 3 Results

Table 2 shows the statistical analysis of MICs and FICs values of chlorhexidine and essential oils. When comparing MICs and FICs data, a significant decrease (*p* ˂ 0.05) was found in the combination of chlorhexidine with cinnamon (mean of MICs 6.00 ± 3.42 mg/mL and FICs of 1.91 ± 1.99 mg/mL), followed by the combination with thyme (mean of MICs 1.98 ± 0.86 mg/mL and FICs 0.50 ± 0.22 mg/mL), oregano (mean of MICs 1.47 ± 0.30 mg/mL and FICs 0.73 ± 0.15 mg/mL) and rosemary (average of MICs 5.73 ± 1.16 mg/mL and FICs 2.62 ± 0.72 mg/mL). For *M. pachydermatis* CBS 1879 strain, a descent in FICs values compared to MICs values is also evident.

**Table 2:** Statistical analysis of MICs and FICs values of chlorhexidine and essential oils in planktonic cells of *Malassezia pachydermatis* (n = 18).

Combined substances	MICs (mg/mL)				FICs (mg/mL)			
	Range	x̅ ± SD	Mo	Me	range	x̅ ± SD	Mo	Me
**Chlorhexidine** **+ clove**	0.08-0.31 3.13-12.5	0.16 ± 0.08 7.29 ± 3.90	0.16 12.5	0.16 6.25	0.01-0.16 1.6-12.5	0.08 ± 0.04 4.70 ± 3.82	0.08 3.13	0.08 3.13
**Chlorhexidine** **+ cinnamon**	0.08-0.31 1.6-12.5	0.16 ± 0.08 6.00 ± 3.42^a^	0.16 6.25	0.16 6.25	0.01-0.16 0.4-6.25	0.08 ± 0.05 1.91 ± 1.99^a^	0.08 1.60	0.08 1.20
**Chlorhexidine** **+ thyme**	0.08-0.31 0.8-3.13	0.16 ± 0.08 1.98 ± 0.86^b^	0.16 1.60	0.16 1.60	0.01-0.08 0.2-0.8	0.06 ± 0.03 0.50 ± 0.22^b^	0.08 0.40	0.08 0.40
**Chlorhexidine** **+ oregano**	0.08-0.31 0.8-1.6	0.16 ± 0.08 1.47 ± 0.30^c^	0.16 1.60	0.16 1.60	0.01 0.4-0.8	0.01 ± 0 0.73 ± 0.15^c^	0.01 0.80	0.01 0.80
**Chlorhexidine** **+ bergamot**	0.04-0.16 6.25-12.5	0.09 ± 0.04 10.42 ± 2.95	0.08 12.5	0.08 12.5	0.01-0.04 6.25-12.5	0.02 ± 0.01 7.29 ± 2.33	0.01 6.25	0.01 6.25
**Chlorhexidine** **+ rosemary**	0.08-0.16 3.13-6.25	0.09 ± 0.03 5.73 ± 1.16^d^	0.08 6.25	0.08 6.25	0.01-0.02 1.6-3.13	0.01 ± 0 2.62 ± 0.72^d^	0.01 3.13	0.01 3.13
***Malassezia pachydermatis* CBS 1879**								
**Chlorhexidine** **+ clove**	0.08 6.25	0.08 ± 0 6.25 ± 0	-	-	0.01 3.13	0.01 ± 0 3.13 ± 0	-	-
**Chlorhexidine** **+ cinnamon**	0.08 6.25	0.08 ± 0 6.25 ± 0	-	-	0.01 6.25	0.01 ± 0 6.25 ± 0	-	-
**Chlorhexidine** **+ thyme**	0.08 0.08	0.08 ± 0 0.08 ± 0	-	-	0.01 0.02	0.01 ± 0 0.02 ± 0	-	-
**Chlorhexidine** **+ oregano**	0.16 1.6	0.16 ± 0 1.6 ± 0	-	-	0.01 0.04	0.01 ± 0 0.04 ± 0	-	-
**Chlorhexidine** **+ bergamot**	0.08 12.5	0.08 ± 0 12.5 ± 0	-	-	0.02 6.25	0.02 ± 0 6.25 ± 0	-	-
**Chlorhexidine** **+ rosemary**	0.08 6.25	0.08 ± 0 6.25 ± 0	-	-	0.01 1.6	0.01 ± 0 1.6 ± 0	-	-

Abbreviations: x̅ – average, SD – standard deviation, Mo – mode, Me – median, MIC – minimal inhibitory concentration, FIC – fractional inhibitory concentration, a-d – mean values with the same superscript letter are statistically significantly different (p˂0.05).

When comparing the means of MICs and FICs in biofilm-forming cells of *M. pachydermatis* (Table 3), a significant decrease (*p* < 0.05) was observed in all tested combinations.

**Table 3:** Statistical analysis of MICs and FICs values of chlorhexidine and essential oils in biofilm-forming cells of *Malassezia pachydermatis* (n = 18).

Combined substances	MICs (mg/mL)				FICs (mg/mL)			
	range	x̅ ± SD	Mo	Me	range	x̅ ± SD	Mo	Me
**Chlorhexidine** **+ clove**	0.08-0.31 3.13-12.5	0.16 ± 0.08 7.29 ± 3.90^a^	0.16 12.5	0.16 6.25	0.01-0.16 1.6-6.25	0.08 ± 0.05 3.14 ± 1.55^a^	0.08 3.13	0.08 3.13
**Chlorhexidine** **+ cinnamon**	0.08-0.31 3.13-12.5	0.16 ± 0.08 7.81 ± 3.49^b^	0.16 6.25	0.16 6.25	0.01-0.16 3.13-6.25	0.08 ± 0.05 4.17 ± 1.47^b^	0.08 3.13	0.08 3.13
**Chlorhexidine** **+ thyme**	0.08-0.31 3.13-6.25	0.16 ± 0.08 4.17 ± 1.47^c^	0.16 3.13	0.16 3.13	0.01-0.16 0.8-3.13	0.08 ± 0.05 1.84 ± 0.97^c^	0.08 1.60	0.08 1.60
**Chlorhexidine** **+ oregano**	0.04-0.31 0.4-0.8	0.17 ± 0.11 0.67 ± 0.19^d^	0.31 0.80	0.12 0.80	0.01-0.16 0.2-0.4	0.07 ± 0.07 0.27 ± 0.09^d^	0.16 0.20	0.05 0.20
**Chlorhexidine** **+ bergamot**	0.04-0.16 1.6-6.25	0.09 ± 0.05 4.44 ± 1.89^e^	0.08 6.25	0.08 4.69	0.01-0.08 0.8-3.13	0.04 ± 0.02 2.23 ± 0.94^e^	0.04 3.13	0.04 2.37
**Chlorhexidine** **+ rosemary**	0.04-0.16 0.8-3.13	0.09 ± 0.05 1.59 ± 0.77^f^	0.04 1.6	0.06 1.60	0.02-0.08 0.4-1.6	0.04 ± 0.03 0.67 ± 0.44^f^	0.02 0.40	0.03 0.40
***Malassezia pachydermatis* CBS 1879**								
**Chlorhexidine** **+ clove**	0.08 6.25	0.08 ± 0 6.25 ± 0	-	-	0.01 3.13	0.01 ± 0 3.13 ± 0	-	-
**Chlorhexidine** **+ cinnamon**	0.08 6.25	0.08 ± 0 6.25 ± 0	-	-	0.01 3.13	0.01 ± 0 3.13 ± 0	-	-
**Chlorhexidine** **+ thyme**	0.08 3.13	0.08 ± 0 3.13 ± 0	-	-	0.01 1.6	0.01 ± 0 1.6 ± 0	-	-
**Chlorhexidine** **+ oregano**	0.04 0.8	0.04 ± 0 0.8 ± 0	-	-	0.01 0.04	0.01 ± 0 0.04 ± 0	-	-
**Chlorhexidine** **+ bergamot**	0.04 1.6	0.04 ± 0 1.6 ± 0	-	-	0.01 0.8	0.01 ± 0 0.8 ± 0	-	-
**Chlorhexidine** **+ rosemary**	0.04 3.13	0.04 ± 0 3.13 ± 0	-	-	0.02 1.6	0.02 ± 0 1.6 ± 0	-	-

Abbreviations: x̅ – average, SD – standard deviation, Mo – mode, Me – median, MIC – minimal inhibitory concentration, FIC – fractional inhibitory concentration, a-e – mean values with the same superscript letter are statistically significantly different (p˂0.05).

The statistical analysis of FICI values is presented in Table 4. No significance was found when comparing the effect of chlorhexidine and essential oils tested on planktonic and biofilm-forming cells of *M. pachydermatis*. For the tested isolates, the best combination appears to be chlorhexidine with oregano (mean of FICI in planktonic cells 0.54 ± 0.02 and in biofilm-forming cells 0.77 ± 0.18) and bergamot (mean of FICI in planktonic cells 0.94 ± 0.28 and in biofilm-forming cells 0.79 ± 0.22).

**Table 4:** Fractional inhibitory concentrations index (FICIs) of chlorhexidine and essential oils combination in *Malassezia pachydermatis* planktonic and biofilm-forming cells.

Combined substances	Planktonic cells				Biofilm-forming cells			
	range	x̅ ± SD	Mo	Me	range	x̅ ± SD	Mo	Me
**Chlorhexidine** **+ clove**	1.00-1.50	1.11 ± 0.18	1.01	1.01	0.38-1.01	0.90 ± 0.23	1.0	1.0
**Chlorhexidine** **+ cinnamon**	0.56-1.25	0.78 ± 0.22	0.76	0.76	0.63-1.50	1.06 ± 0.34	1.0	1.0
**Chlorhexidine** **+ thyme**	0.25-1.25	0.71 ± 0.33	-	0.63	0.38-1.50	0.90 ± 0.34	-	0.88
**Chlorhexidine** **+ oregano**	0.52-0.56	0.54 ± 0.02	0.56	0.55	0.50-1.00	0.77 ± 0.18	1.0	0.75
**Chlorhexidine** **+ bergamot**	0.63-1.25	0.94 ± 0.28	0.63	0.94	0.50-1.00	0.79 ± 0.22	1.0	0.88
**Chlorhexidine** **+ rosemary**	0.38-0.64	0.59 ± 0.09	0.63	0.63	0.75-1.01	0.92 ± 0.12	1.0	1.0
***Malassezia pachydermatis* CBS 1879**								
**Chlorhexidine** **+ clove**	0.63	-	-	-	0.63	-	-	-
**Chlorhexidine** **+ cinnamon**	1.10	-	-	-	0.63	-	-	-
**Chlorhexidine** **+ thyme**	0.38	-	-	-	0.64	-	-	-
**Chlorhexidine** **+ oregano**	0.28	-	-	-	0.75	-	-	-
**Chlorhexidine** **+ bergamot**	0.75	-	-	-	0.75	-	-	-
**Chlorhexidine** **+ rosemary**	0.38	-	-	-	1.01	-	-	-

Abbreviations: x̅ – average, SD – standard deviation, Mo – mode, Me – median, MIC – minimal inhibitory concentration, FICI – fractional inhibitory concentration index.

**Fig. 1:**
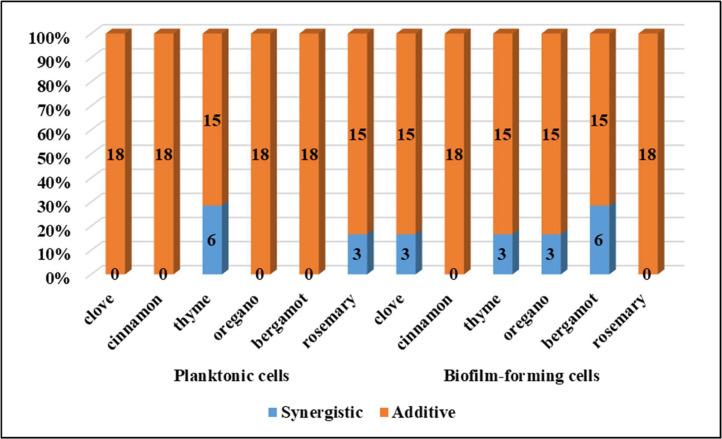
The effect of the combination of chlorhexidine with essential oils on planktonic and biofilm-forming cells of *Malassezia pachydermatis*.

Evaluation of the effect of chlorhexidine in combination with essential oils in planktonic and biofilm-forming cells of *M. pachydermatis* based on FICI values is documented in Table 5 and shown in Fig. 1. In planktonic cells, most combinations exhibited the additive effect. A synergistic effect was noted when chlorhexidine was combined with thyme (33.3 %) or rosemary (16.7 %). Similarly, in the biofilm-forming cells, the additive effect was often detected, but the combination of chlorhexidine with clove, thyme, oregano and bergamot produced synergistic effect. In the planktonic cells of *M. pachydermatis* CBS 1879, a synergistic effect was noted in the combinations of chlorhexidine with thyme, oregano and rosemary (see FICI in Table 4), however in the biofilm-forming cells only additive effect was found in all tested combinations.

**Table 5:** Evaluation of the effect (n/%) of chlorhexidine with essential oils on the planktonic and biofilm-forming cells of *Malassezia pachydermatis*.

Effect	Planktonic cells					
	Clove	Cinnamon	Thyme	Oregano	Bergamot	Rosemary
	n/%	n/%	n/%	n/%	n/%	n/%
**Synergistic**	0/0	0/0	6/33.3	0/0	0/0	3/16.7
**Additive**	18/100	18/100	12/66.7	18/100	18/100	15/83.3

Effect	Biofilm-forming cells					
	Clove	Cinnamon	Thyme	Oregano	Bergamot	Rosemary
	n/%	n/%	n/%	n/%	n/%	n/%
**Synergistic**	3/16.7	0/0	3/16.7	3/16.7	6/33.3	0/0
**Additive**	15/83.3	18/100	15/83.3	15/83.3	12/66.7	18/100

## 4 Discussion

Due to the increasing resistance to antifungal medications, the therapy of yeast infection is an increasing problem. For the control and initial treatment of *Malassezia* dermatitis, antiseptic substances are recommended, especially chlorhexidine, which is part of several cosmetic shampoos (at a concentration of 2–3 %) or disinfectants, usually combined with azole antifungals (Maynard et al., 2011).

In our study, the potential *in vitro* synergistic effect of selected essential oils and chlorhexidine on planktonic and biofilm-forming cells of *M. pachydermatis*, was investigated.

Several authors report the inhibitory effect of essential oils alone or in combination with antifungal drugs on *Malassezia* species, but data on the effectiveness of combinations of EOs with other potential antifungal agents are very rare.

The antifungal activity of 22 EOs against 15 clinical isolates of *M. pachydermatis* was determined by Bismarck et al. (2019). The strong effect of EOs of winter savory (*Satureja montana* L.), lemon grass (*Cymbopogon citratus* (DC.) STAPF), oregano (*Origanum vulgare* L.), palmarosa (*Cymbopogon martinii* (ROXB.) J.F.WATSON), and cinnamon leaf (*Cinnamomum verum* J.S. PRESL) was demonstrated. Oregano and thyme EO and their phenolic compounds (carvacrol and thymol) were also found to have fungicidal efficacy against *M. pachydermatis* (Sim et al., 2019). Schlemmer et al. (2019) studied *in vitro* activities of EO components (carvacrol, cinnamaldehyde and thymol) alone and in combination with antifungal agents (fluconazole, itraconazole, ketoconazole, clotrimazole, miconazole, terbinafine and nystatin) against *M. pachydermatis*. The mean fractional inhibitory concentration index (FICI) showed synergies for the combinations of nystatin with carvacrol, nystatin with thymol, and miconazole with carvacrol. The synergistic interactions between clotrimazole and tea-tree oil (*Melaleuca alternifolia*), peppermint (*Mentha piperita*) and oregano EO were reported by Bohmova et al. (2019).

Since we have not yet found any article dealing with the effectiveness of the combination of chlorhexidine and essential oil, we decided to set out such an experiment.

Although no significant difference was found when comparing FICI means in planktonic and biofilm-forming cells, statistical significance was detected when comparing MICs means to FICs means of chlorhexidine and tested essential oils in both planktonic and biofilm-forming cells.

According to our findings, oregano EO was most effective on planktonic cells (MIC 1.47 ± 0.30 mg/mL) and biofilm-forming cells (0.67 ± 0.19 mg/mL) of *M. pachydermatis*. The combination with chlorhexidine caused a decrease to 0.73 ± 0.15 mg/mL and 0.27 ± 0.09 mg/mL respectively. A much higher MIC for oregano EO (6.76 – 7.73 mg/mL) against *M. pachydermatis* was reported by Ebani et al. (2020). Vinciguerra et al. (2019) found out the effective MIC for oregano EO in *M. furfur* at 780 µg/mL. In *Candida albicans*, the effect of oregano EO is explained by the content of active substances that bind to sterols in the fungal membranes (Lima et al., 2013). According to Ultee et al. (1999), carvacrol, one of the main component in oregano EO, interacts with cell membranes by changing the permeability for small cations (Ultee et al., 1999). We assume that similar mechanism of action also affects the cell membrane in *M. pachydermatis*.

The data in our study also show high activity of thyme essential oil against *M. pachydermatis* isolates. The MIC achieved 1.98 ± 0.86 mg/mL in planktonic cells and 4.17 ± 1.47 mg/mL in biofilm-forming cells. Also in this case, the combination with chlorhexidine led to the decrease in concentrations (FIC = 0.50 ± 0.22 mg/mL and 1.84 ± 0.97 mg/mL respectively). In the study of Ebani (2020), the MIC of thyme EO ranged from 7.73 to 8.7 mg/mL, which is a much higher concentration compared to our results. According to our knowledge, this can be influenced by the different content of the active compounds in EO. The major active compound of thyme is thymol, which exerts its antimicrobial action via binding to membrane proteins through hydrophobic and hydrogen bonds, and subsequently changing the permeability of cell membranes (Burt, 2004).

Rosemary EO also appears to be very effective against several *Malassezia* species. Khosravi et al. (2016) found that many pathogenic *Malassezia* isolates were susceptible to rosemary EO (*M. furfur* at 26 µg/mL, *M. slooffiae* at 250 µg/mL, *M. sympodialis* at 420 µg/mL, *M. obtusa* at 410 µg/mL, *M. globosa* at 850 µg/mL, *M. nana* at 100 µg/mL and *M. restricta* at 350 µg/mL). Similarly, our findings indicate susceptibility of *M. pachydermatis* to rosemary EO at low concentrations. As an agent tested alone, rosemary EO inhibited the growth of planktonic cells at the MIC of 5.73 ± 1.16 mg/mL and in biofilm-forming cells at the MIC of 1.59 ± 0.77 mg/mL. The combination with chlorhexidine resulted in the decrease of concentrations to 2.62 ± 0.72 mg/mL and 0.67 ± 0.44 mg/mL respectively. Cineole is the main active component of rosemary EO and its antimicrobial action, like other EOs, is thought to be due to its ability to alter the membrane and cell wall, leading to the extracellular loss of cytoplasmic material (Altintas et al., 2013).

In this study, the highest inhibitory effect was observed in cinnamon EO, where when tested alone, the mean of MIC reached 6.00 ± 3.42 mg/mL in planktonic cells and 7.81 ± 3.49 mg/mL in biofilm-forming cells, but in combination with chlorhexidine, the FIC values were 1.91 ± 1.99 mg/mL and 4.17 ± 1.47 mg/mL respectively. Ebani et al. (2020) refer the effect of cinnamon EO against *M. pachydermatis* at the MIC of 3.06 – 4.08 mg/mL. Cinnamon EO was also found to be active against *M. furfur* at a concentration of 32 µg/mL (Pooja et al., 2013). The main component of cinnamon, cinnamaldehyde, is responsible for the antimicrobial activity in microorganisms by inhibiting cell wall biosynthesis, membrane function, and specific enzyme activities (Shreaz et al., 2016).

Higher concentrations of clove essential oil were required for inhibition of planktonic cells growth and biofilm disintegration (both MIC of 7.29 ± 3.90 mg/mL). When combined with chlorhexidine, a decrease in concentrations was noted (FIC of 4.70 ± 3.82 mg/mL and 3.14 ± 1.55 mg/mL respectively). Shahi et al. (2015) report the effectiveness of clove EO also against *M. furfur* isolates at a concentration of 0.625 µL/mL. The essential oil of clove is able to destroy the cell walls and membranes of microorganisms, penetrate through cytoplasmic membranes or enter the cells, and subsequently inhibit the normal synthesis of DNA and proteins (Xu et al., 2016).

Out of the six EOs tested, bergamot EO showed the lowest antifungal and antibiofilm activity, with an average MIC of 10.42 ± 2.95 mg/mL for planktonic cells and 4.4 ± 1.89 mg/mL for biofilm-forming cells. When combined with chlorhexidine, FIC values were 7.29 ± 2.33 mg/mL in planktonic cells and 2.23 ± 0.94 mg/mL in biofilm-forming cells. Nevertheless, in combination with chlorhexidine, an additive effect was registered in all tested planktonic cells (100 %) and in biofilm-forming cells, a synergistic effect was found in 6 isolates (33.3 %) and the additive effect in 12 isolates (66.7 %).

The *in vitro* antifungal activity of three bergamot oils (natural essence, furocoumarin-free extract and distilled extract) tested alone and in combination with the antiseptic, boric acid, against *Candida* species, was investigated by Romano et al. (2005). In all tested *Candida* spp., the MIC90 for all bergamot oils in combination with boric acid were significantly lower than the corresponding values for the oils alone (*p* < 0.05). The most abundant compounds, limonene and linalool, are responsible for the synergistic effect of antimicrobial activity, by the ability to alter the integrity of the cell wall (Quirino et al., 2022).

Despite the fact that the use of plant essential oils for the treatment of superficial *Malassezia* infections doesn't currently seem to be the first choice, several preparations intended for the treatment of the skin contain, in addition to an antiseptic (e.g. chlorhexidine), also some plant component – either an extract or an essential oil. Although the initial intention of the manufacturers could have been to make the preparation more attractive (improve the smell), the presence of a plant extract or oil can likely potentiate its antifungal effect.

## 5 Conclusion

The results from this study have shown that the use of one of the tested plant essential oils – oregano, rosemary, thyme, clove, cinnamon, or bergamot simultaneously with the antiseptic substance chlorhexidine can improve their effect on planktonic as well as biofilm-forming cells of *M. pachydermatis*. Based on the evaluation of the fractional inhibitory concentration index, our study confirmed that the use of chlorhexidine combined with one of the mentioned essential oils allows to reduce the concentration of individual compounds while maintaining sufficient antifungal effectiveness. This may be helpful in adjunctive therapy or prophylaxis of *Malassezia* skin and ear infections in dogs as well as in the prevention of resistance to the antifungal drugs.

## Ethical statement

The work does not involve the use of animal subjects.

## CRediT authorship contribution statement

**Peter Váczi:** Writing – original draft, Methodology, Investigation, Conceptualization. **Eva Čonková:** Methodology, Investigation. **Zuzana Malinovská:** Visualization, Formal analysis.

## Declaration of competing interest

The authors declare that they have no known competing financial interests or personal relationships that could have appeared to influence the work reported in this paper.
